# Current Trends in Autologous Breast Reconstruction and the Implications of Impending Changes to Insurance Reimbursement

**DOI:** 10.7759/cureus.43855

**Published:** 2023-08-21

**Authors:** Arman J Fijany, Nicole Friedlich, Sofia E Olsson, Anthony E Bishay, Maxim Pekarev

**Affiliations:** 1 Surgery, Vanderbilt University Medical Center, Nashville, USA; 2 Anne Burnett Marion School of Medicine, Texas Christian University, Fort Worth, USA; 3 Neurosurgery, Vanderbilt University School of Medicine, Nashville, USA

**Keywords:** socioeconomic disparities, insurance reimbursement, s-codes, diep flap, autologous breast reconstruction

## Abstract

Introduction

In 2019, the Centers for Medicare & Medicaid Services (CMS) combined all autologous breast flap procedures under one billing code, effective from December 31, 2024. This change will result in equal insurance reimbursement rates for popular flap options, such as transverse rectus abdominis muscle (TRAM) and deep inferior epigastric perforator (DIEP) flaps, which were previously billed separately using S-codes based on complexity.

Methods

This study aimed to analyze insurance code changes for autologous breast reconstruction flap procedures. Data were collected from the American Society of Plastic Surgeons' annual plastic surgery statistics reports, including specific insurance codes and case volumes from 2007 to 2020. A comprehensive analysis was conducted to assess recent trends in flap utilization rates, documenting any modifications or additions to the existing codes and their implementation years.

Results

The study analyzed billing codes and case volumes for autologous breast reconstruction procedures, with a focus on the DIEP flap and other alternatives. Non-autologous breast reconstruction procedures showed consistently higher case volumes compared to autologous procedures from 2007 to 2020. Notably, the popularity of the DIEP flap surpassed that of other flap options after 2011.

Conclusion

The removal of S-codes for autologous breast reconstruction by CMS and the subsequent potential decrease in insurance coverage for the DIEP flap may lead to a decrease in its utilization and a shift toward more invasive options, like the TRAM flap. This change could result in financial burdens for patients and widen socioeconomic disparities in breast reconstruction, limiting access to preferred reconstructive methods and impacting patient autonomy and overall well-being.

## Introduction

In 2019, the Centers for Medicare & Medicaid Services (CMS) implemented a protocol to combine all autologous breast flap procedures under a single billing code, 193464 [1]. Under this protocol, popular reconstructive flap options, such as the transverse rectus abdominis muscle (TRAM) and deep inferior epigastric perforator (DIEP) flap, would be billed using the same medical code and provide the same insurance reimbursement rate. These changes are set to take effect after December 31, 2024.

Currently, most plastic surgeons receive reimbursement from insurance companies for specific flap reconstruction procedures with S-codes, such as S2068 for DIEP flap surgery, S2066 for the superior gluteal artery perforator (SGAP) flap surgery, and S2067 for stacked flap surgery, where multiple flaps are stacked on top of one another to accommodate for lack of flap volume with a single flap [2]. S-code reimbursement is greater for perforator flaps than non-perforator flaps due to the increased complexity of dissection, cost, and time required [3]. This difference in reimbursement reflects the intricate nature of perforator flap dissections, the higher costs associated with specialized equipment and imaging techniques, as well as the extended operative time. Notably, it is important to recognize that the complexity extends beyond perforator and non-perforator flaps, as procedures like TRAM flaps used in breast reconstruction also contribute their unique set of challenges to the overall spectrum of complexities in reconstructive surgery [3].

## Materials and methods

Data collection

The primary objective of this study was to comprehensively analyze insurance code changes concerning flap procedures for autologous breast reconstruction. To achieve this, we employed a meticulous data collection approach that involved gathering relevant information from multiple reliable sources.

Sources of data

We collected the necessary data from the annual plastic surgery statistics reports, which are published by the esteemed American Society of Plastic Surgeons [4]. These reports contain comprehensive information on various plastic surgery procedures performed over the years, providing a valuable resource for our study.

Insurance code identification

To ensure a robust analysis, we first identified and compiled a comprehensive list of specific insurance codes associated with autologous breast reconstruction flap procedures [2]. This list included detailed information about each code, such as its purpose and relevance to the procedures under investigation.

Case volume records

The case volume data for the autologous flap procedures were meticulously gathered from the annual plastic surgery statistics report [5]. These case volumes represent the total number of times each autologous flap procedure was performed within a specified time frame. For our study, we focused on a year-to-year comparison from 2007 to 2020 to provide a comprehensive overview of the trends in flap utilization rates for breast reconstruction.

Data analysis

After obtaining the insurance codes and corresponding case volumes, we performed a detailed analysis to assess the impact of insurance code changes on the utilization of the DIEP flap procedure. First, we identified instances of insurance code changes related to the DIEP flap procedure by comparing the codes recorded in consecutive annual plastic surgery reports. Any modifications or additions to the existing codes were noted, and the corresponding years of implementation were documented. To examine the effect of code changes on the utilization of the DIEP flap, we compared the case volumes before and after each identified code change. This allowed us to assess any fluctuations in procedure frequency and identify potential trends related to insurance code modifications.

## Results

Billing codes assessed through the annual plastic surgery statistics report

The analysis focused on the insurance codes and case volumes associated with the DIEP flap procedure and other autologous flap alternatives, as obtained from the CMS [2]. Table 1 presents the billing codes assessed in this study, along with the specific procedure for each code.

**Table 1:** Commonly used billing codes in autologous breast reconstruction and their corresponding procedures. TRAM: transverse rectus abdominis muscle; DIEP: deep inferior epigastric perforator; SIEA: superficial inferior epigastric artery; GAP: gluteal artery perforator.

Billing code for microsurgical breast reconstruction	Procedure performed
19364	Breast reconstruction; with any free flap (e.g., TRAM, DIEP, SIEA, GAP flap).
S2066	Breast reconstruction with gluteal artery perforator (GAP) flap, including flap harvesting, microvascular transfer, closure of donor site, and shaping the flap into a breast, unilateral.
S2067	Breast reconstruction of a single breast with "stacked" deep inferior epigastric perforator (DIEP) flap(s) and/or gluteal artery perforator (GAP) flap(s), including harvesting of the flap(s), microvascular transfer, closure of donor site(s), and shaping the flap into a breast, unilateral.
S2068	Breast reconstruction with deep inferior epigastric perforator (DIEP) flap or superficial inferior epigastric artery (SIEA) flap, including flap harvesting, microvascular transfer, closure of donor site, and shaping the flap into a breast, unilateral.

Case volume of autologous and non-autologous breast reconstruction

**Figure 1:**
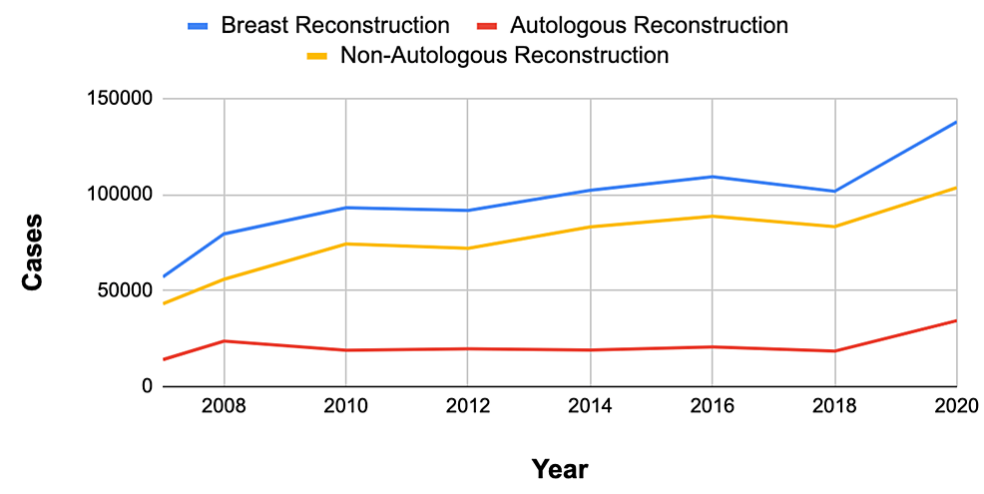
Total breast reconstruction, autologous reconstruction, and non-autologous reconstruction cases. Trends in autologous and non-autologous breast reconstruction (2008-2020).

Non-autologous breast reconstruction procedures had a higher case volume (603,593 cases) compared to autologous breast reconstruction procedures (168,652 cases) (Table 2). This pattern was persistent from 2007 to 2020 (Figure 1).

**Table 2:** Data on autologous and non-autologous breast reconstruction case volume published by the American Society of Plastic Surgeons.

Year	Breast reconstruction cases	Autologous cases	Non-autologous cases	Percent autologous	Increase in autologous case volume from the prior report
2020	137,808	34,323	103,485	24.9%	1.86
2018	101,657	18,441	83,216	18.1%	0.89
2016	109,256	20,650	88,606	18.9%	1.08
2014	102,215	19,066	83,149	18.7%	0.97
2012	91,655	19,643	72,012	21.4%	1.04
2010	93,083	18,888	74,195	20.3%	0.80
2008	79,458	23,619	55,839	29.7%	1.86
2007	57,102	14,022	43,091	24.6%	0.89

Case volume for autologous breast reconstruction procedures

**Figure 2:**
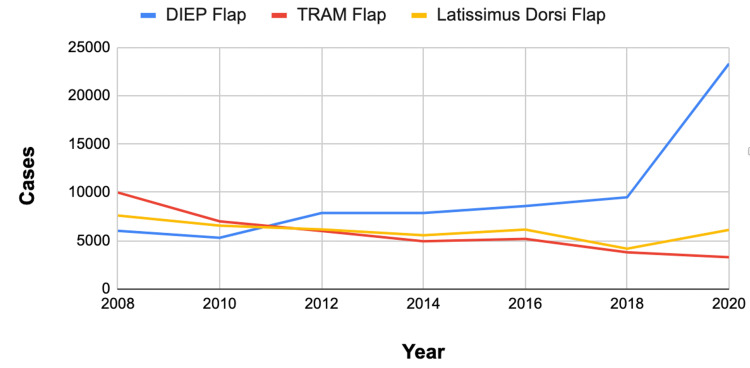
DIEP, TRAM, and latissimus dorsi flap utilization in breast reconstruction. Trends in procedures for autologous breast reconstruction (2008-2020). TRAM: transverse rectus abdominis muscle; DIEP: deep inferior epigastric perforator.

Until 2011, the case volume for the DIEP flap procedure was consistently lower than that of both the TRAM flap and latissimus dorsi flap procedures (Table 3). However, starting in 2011, there was a notable shift in popularity, and the case volume for the DIEP flap exceeded that of the TRAM flap and latissimus dorsi flap (Figure 2).

**Table 3:** Data on the case volume for common autologous flaps used in breast reconstruction published by the American Society of Plastic Surgeons. TRAM: transverse rectus abdominis muscle; DIEP: deep inferior epigastric perforator.

Year	DIEP flap	TRAM flap	Latissimus flap	Other flaps
2020	23,324	3,297	6,128	1,574
2018	9,497	3,799	4,188	957
2016	8,585	5,190	6,151	724
2014	7,866	4,939	5,572	689
2012	7,866	6,007	6,173	937
2010	5,308	7,009	6,571	Not recorded
2008	6,018	9,987	7,614	Not recorded

Benefits and short-comings of commonly used flap procedures

We also evaluated and compared the benefits and shortcomings of commonly used flap procedures for breast reconstruction. The flap procedures analyzed included TRAM, gluteal artery perforator (GAP), "stacked" flaps, DIEP, and superficial inferior epigastric artery (SIEA) flaps. The findings are summarized in Table 4.

**Table 4:** Comparison of commonly used flaps in autologous breast reconstruction. TRAM: transverse rectus abdominis muscle; DIEP: deep inferior epigastric perforator; SIEA: superficial inferior epigastric artery; GAP: gluteal artery perforator.

Flap	Pros and cons
TRAM	
Pros:	· Long pedicle length allows the flap to be used as a pedicled or free flap
	· Easier dissection
	· Additional volume compared to perforator flaps (DIEP)
	· Decreased incidence of partial and full-thickness fat necrosis when compared to DIEP flaps
Cons:	· Involves abdominal muscle, which results in a high hernia risk post-operatively and subsequent core muscle weakness
	· More painful operation
	· Cannot be done in patients with previous abdominoplasty
GAP	
Pros:	· Hidden donor site
	· An option for patients with a paucity of abdominal tissue
	· Can be done in patients with previous abdominoplasty
Cons:	· Burdensome dissection
	· Gluteal fat is not easily manipulated
	· Can result in a contour irregularity of the buttocks
“Stacked”	
Pros:	· Can be done in women with an extreme paucity of autologous tissue options
	· Provides the most significant amount of breast volume
Cons:	· Tedious operation (multiple free flaps involved)
	· Increased donor site morbidity and flap complications due to additional tissue/anastomosis
DIEP	
Pros:	· Less comparative risk of hernia
	· Improved outcomes, both in terms of patient satisfaction and complication rate
	· Improved abdominal contour
	· Less costly procedure
Cons:	· Requires additional skill to identify perforator, tedious dissection
	· Minimal pedicle length
	· Less reliable blood supply
	· Cannot be done in patients with previous abdominoplasty
	· Some women might not have adequate volume
	· High incidences of partial and full-thickness fat necrosis
SIEA	
Pros:	· Incurs minor damage to the abdominal muscles (less than DIEP flap)
	· Minimal risk of hernia post-operatively
	· Improved abdominal contour
Cons:	· Vessels are the least reliable and often need better quality or better visualization for free flap transfer
	· Smaller in volume
	· Most challenging vessel dissection to perform technically
	· Highest rates of fat necrosis and flap failure

## Discussion

In 2021, the CMS discontinued S-codes for autologous breast reconstruction, making the 193464 billing code the sole source of insurance reimbursement for reconstructive breast procedures. In anticipation of these changes, several insurance companies have pre-emptively ceased coverage for the DIEP flap under S2068; other insurance companies are expected to follow [6].

With the removal of appropriate additional reimbursement for the DIEP flap procedure, there is concern that breast reconstruction surgeons will decrease their DIEP flap case volume and potentially revert to utilizing more invasive autologous options, such as the TRAM flap [3,7]. While the DIEP flap procedure is associated with fewer long-term complications than the TRAM flap procedure, it is more costly and time-consuming for most plastic surgeons [3]. However, though the complexity of DIEP-based reconstruction was a noticeable barrier to care in the past, the DIEP flap is now considered the gold standard in autologous breast reconstruction [8] and certain surgeons can perform the procedure in an extremely efficient manner [9,10]. While TRAM-based breast reconstruction can be performed by a more significant proportion of reconstructive surgeons, the procedure compromises abdominal musculature, leaving patients with significant morbidity and an increased risk of bowel herniation [3]. The muscle-sparing version of the TRAM flap is associated with a decreased risk of hernia formation [11]; however, there is still a risk for herniation that can be avoided entirely with DIEP flaps. Other popular flap options have their benefits and downfalls; however, the DIEP flap is currently considered a superior option for breast reconstruction (Table 2) [3]. Importantly, the average surgical duration for DIEP flap procedures has been steadily decreasing, with many surgeons now being able to perform the surgeries as expeditiously as TRAM flap reconstruction.

There has been an increase in the frequency of DIEP flaps performed following the introduction of S-codes [12]. This trend is likely due to the fact that a specific S-code may facilitate billing and reimbursement processes for healthcare providers [13]. Furthermore, along with incentivizing surgeons to more frequently perform DIEP flaps, S-codes allow for better documentation and tracking of procedures performed. It is likely that many autologous reconstructive procedures, especially DIEP, will risk de-incentivization following the removal of specific S-codes.

Without the additional reimbursement provided by S-codes, there is fear that surgeons cannot cover the DIEP procedure costs [14]. For the patients that prefer DIEP flap-based reconstruction, many will potentially have to pay additional out-of-pocket costs or settle with less admired reconstruction options. Many women already pay over $5,000 out-of-pocket for their autologous reconstruction procedure [15]. With an increased financial pressure to turn to inferior reconstructive procedures, the burden on these patients is increased, many of whom just began dealing with significant stress after a breast cancer diagnosis. A patient’s choice of breast reconstruction method is a deeply personal and challenging decision. There is broad support from the medical community for patients to be able to choose a surgery that is the best fit for them [7,16]. All appropriate surgical and medical options must be offered to serve patients best and provide autonomy. This is vital in shared decision-making and avoiding unwanted complications like lymphedema.

Furthermore, these insurance changes may broaden the socioeconomic disparities in breast reconstruction [17]. Patients with the means to afford high out-of-pocket costs will be more likely to receive superior breast reconstructions than those under contracts of commercial insurance plans. Insurance coverage under these new changes resembles coverage rates in the 1980s, where individuals requiring breast reconstruction had limited access to reconstructive options [18].

Sociodemographic factors currently represent disparities in autologous breast reconstruction and implant-based reconstruction [19,20]. Patients with private insurance were more likely to have autologous reconstruction than those with Medicare. Medicare and Medicaid patients are also less likely to pursue any form of breast reconstruction following mastectomy [21].

Implant-based reconstruction is another standard method for breast reconstruction. Some individuals prefer autologous tissue options due to their more natural feel and appearance. Additionally, increased reports and awareness of breast implant illness (BII) and breast implant-associated anaplastic large cell lymphoma (BIA-ALCL) has decreased interest in implant-based reconstructive options [22]. The decision to pursue breast reconstruction, as well as which method of reconstruction to use, is incredibly personal and must be made between patients and their providers. Discussions within the patient-physician relationship should be based on patient preferences for surgical outcomes, not cost.

Limitations

Despite the rigorous data collection and analysis, certain limitations must be acknowledged. The reliance on aggregated case volume data from annual reports might have introduced sampling bias, and the lack of individual patient data limited our ability to explore patient-specific factors. Additionally, the focus on a specific time frame (2007 to 2020) and the potential variability in insurance coding practices should be considered when interpreting the results. Nonetheless, our research serves as a foundation for further exploration and underscores the importance of monitoring insurance code updates in the realm of plastic surgery practices.

## Conclusions

Limiting access to alternative autologous reconstructive options for those recently afflicted by a life-threatening breast cancer diagnosis revokes patient autonomy and heightens socioeconomic health disparities. Introducing and popularizing the DIEP flap was a productive step forward in the field of breast reconstruction. In recent years, the usage and popularity of the DIEP flap have skyrocketed; however, recent changes in reimbursement can potentially result in an inflection point in the number of cases performed in the future. Limiting access to this option only to those who can pay out-of-pocket may dramatically impact our patients' physical, mental, and overall well-being.
